# Delineating managed land for reporting national greenhouse gas emissions and removals to the United Nations framework convention on climate change

**DOI:** 10.1186/s13021-018-0095-3

**Published:** 2018-05-29

**Authors:** Stephen M. Ogle, Grant Domke, Werner A. Kurz, Marcelo T. Rocha, Ted Huffman, Amy Swan, James E. Smith, Christopher Woodall, Thelma Krug

**Affiliations:** 1 Natural Resource Ecology Laboratory and Department of Ecosystem Science and Sustainability, Colorado State University, Fort Collins, CO 80523 USA; 2 USDA Forest Service, Northern Research Station, 1992 Folwell Avenue, St. Paul, MN 55108 USA; 3 Natural Resources Canada, Canadian Forest Service, 506 West Burnside Road, Victoria, BC V8Z 1M5 Canada; 4 Fábrica Éthica Brasil, Av. Cassatella 177, Jundiaí, 13218-55 Brazil; 5 Agriculture and Agri-Food Canada, 960 Carling Ave, Ottawa, ON K1A 0C6 Canada; 6 Natural Resource Ecology Laboratory, Colorado State University, Fort Collins, CO 80523 USA; 7 USDA Forest Service, Northern Research Station, Forest Sciences Laboratory, 271 Mast Road, Durham, NH 03824 USA; 8 National Institute for Space Research, Ministry of Science, Technology, Innovation and Communication (INPE/MCTIC), São José dos Campos, São Paulo, Brazil

**Keywords:** Greenhouse gas emissions inventory, Carbon inventory, Managed land proxy, Land use, Agriculture, Forestry

## Abstract

Land use and management activities have a substantial impact on carbon stocks and associated greenhouse gas emissions and removals. However, it is challenging to discriminate between anthropogenic and non-anthropogenic sources and sinks from land. To address this problem, the Intergovernmental Panel on Climate Change developed a managed land proxy to determine which lands are contributing anthropogenic greenhouse gas emissions and removals. Governments report all emissions and removals from managed land to the United Nations Framework Convention on Climate Change based on this proxy, and policy interventions to reduce emissions from land use are expected to focus on managed lands. Our objective was to review the use of the managed land proxy, and summarize the criteria that governments have applied to classify land as managed and unmanaged. We found that the large majority of governments are not reporting on their application of the managed land proxy. Among the governments that do provide information, most have assigned all area in specific land uses as managed, while designating all remaining lands as unmanaged. This designation as managed land is intuitive for croplands and settlements, which would not exist without management interventions, but a portion of forest land, grassland, and wetlands may not be managed in a country. Consequently, Brazil, Canada and the United States have taken the concept further and delineated managed and unmanaged forest land, grassland and wetlands, using additional criteria such as functional use of the land and accessibility of the land to anthropogenic activity. The managed land proxy is imperfect because reported emissions from any area can include non-anthropogenic sources, such as natural disturbances. However, the managed land proxy does make reporting of GHG emissions and removals from land use more tractable and comparable by excluding fluxes from areas that are not directly influenced by anthropogenic activity. Moreover, application of the managed land proxy can be improved by incorporating additional criteria that allow for further discrimination between managed and unmanaged land.

## Background

Greenhouse gas (GHG) emissions from land use are a substantial contributor to global emissions, particularly carbon (C) stock changes from land-use conversion [1–3]. For example, deforestation, i.e., converting forests to non-forest uses (e.g., settlements) leads to oxidation of organic matter in the tree biomass, litter and dead wood pools both onsite and offsite, and emissions of carbon dioxide (CO_2_) to the atmosphere, as well as emissions of other non-CO_2_ GHGs where burning occurs. Afforestation and reforestation have the potential to reverse this process resulting in a net uptake of CO_2_ from the atmosphere. Such a dynamic was exhibited in the United States during the twentieth century as former croplands were abandoned and reverted to forest in many cases [4]. Land-use conversion also influences soil C pools. For example, cultivation of land leads to a release of soil C ranging from 30 to 60% of the original stock in the topsoil [5, 6]. Adopting conservation tillage, particularly no-till, can increase soil C stocks due to physical protection of organic matter in aggregates [7, 8]. Changing crop production practices, such as planting improved varieties or changing fertilization and irrigation practices can also have an impact on soil C stocks [9]. Land use can have a profound effect on trajectories of terrestrial C sinks and sources, as well as other GHG fluxes, and consequently, quantifying the impacts of land use and land-use change is a fundamental component of national GHG inventories.

The Intergovernmental Panel on Climate Change (IPCC) has developed guidelines for estimating anthropogenic GHG emissions and removals [9–11] that are used to compile national GHG inventories for reporting to the United Nations Framework Convention on Climate Change (UNFCCC). National greenhouse gas inventories focus on anthropogenic sources of emissions because policy interventions directly influence human activities contributing to emissions. Other, non-anthropogenic sources of emissions, such as permafrost melting in the arctic and associated CO_2_ fluxes, are important for understanding the global C cycle, but are less likely to be impacted by direct management actions. In the case of permafrost, indirect effects are possible by reducing direct emissions from anthropogenic sources, such as fossil fuel combustion, fertilization management for croplands, waste management, but direct management of permafrost seems unlikely. Therefore, as part of the IPCC guidelines, GHG emissions and associated C stock changes from land use and management activities are to be estimated for all managed land in the country.

The IPCC defines managed land as “… land where human interventions and practices have been applied to perform production, ecological or social functions” [9]. However, the use of “managed land” as a proxy for anthropogenically-driven GHG fluxes does not always require active management such as in cropland, settlements or commercial forest land. It may also incorporate management decisions that are more nuanced, such as protecting lands from wildfires, using wilderness for recreation or designating areas for conservation. The IPCC guidance provides latitude for governments to refine the definition of managed land to meet their national circumstances, but the definition should be applied consistently over time and across the territory in the country. Reporting should also be transparent and include descriptions of the methods and definitions used to determine areas of managed and unmanaged lands. Determining the managed land base will have implications for C stock changes and associated GHG emissions that are reported to the UNFCCC and addressed by policy actions to mitigate GHG emissions.

Emissions and removals of GHGs from the land surface occur even without anthropogenic interventions through land management. For example, forests accumulate and lose C through production and decomposition processes, and these trajectories can change with natural disturbances [12]. In addition, there is a limit to how much a land manager can influence GHG emissions on managed land because the processes driving C stock changes and other GHG fluxes are not fully controlled by the manager. This has been recognized in the agreements for the second commitment period of the Kyoto Protocol and participating countries have the option to exclude emissions and subsequent removals from natural disturbances on managed lands with fulfillment of the defined rules [11, 13]. Complete separation of anthropogenic and non-anthropogenic emissions on managed land is not trivial [14, 15], and may not even be feasible. Regardless, the managed land proxy allows inventory compilers to focus on areas in a country directly impacted by management activity, and is considered by the IPCC as the most universally applicable approach for separating anthropogenic and non-anthropogenic emissions associated with land use [14].

Given the disparity in the use of the managed land proxy among UNFCCC member governments, there is a need to understand the concept and associated application by governments. Therefore, our objective is to review steps for delineating a managed land base as a foundation for reporting GHG emissions associated with land use and land-use change. The managed land definition and implementation methods for Brazil, Canada and the United States are examined in more detail. Developing a managed land base involves three general steps, (a) defining managed land taking into consideration national circumstances, (b) developing implementation criteria compatible with the definition, and (c) implementing the analysis to produce the managed land base for GHG reporting.

## Main text

### Use of managed land proxy

**Fig. 1:**
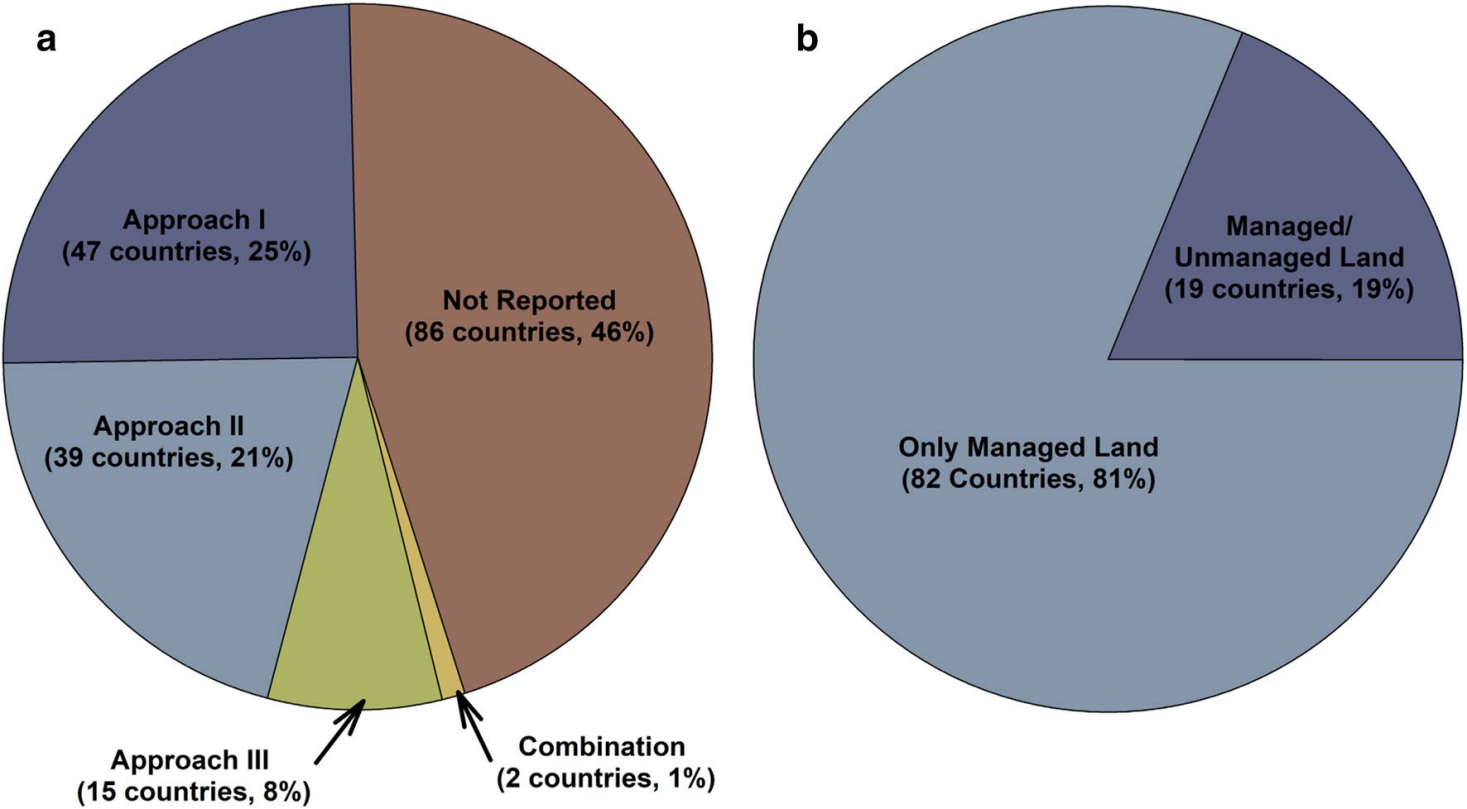
Proportion of governments that use approach I, II or III methods for land representation, or have not included this information in their national communications (IPCC 2003, 2006) (**a**). The subset of governments reporting their approach for land representation that have delineated managed and unmanaged areas in their territories compared to the governments that consider all land as managed (**b**). Data extracted from the latest national communication to the UNFCCC as of 2015 and common reporting format tables submitted in 2018 (http://www.unfccc.int)

While most governments report net emissions from land use based on their national communications to the UNFCCC [16], only 19 countries have delineated managed and unmanaged land areas for their territory based on a review of the latest communications submitted by governments through 2015 and Common Reporting Format (CRF) tables submitted in 2018 (note: CRF tables are only submitted by Annex I parties to the convention) (Fig. 1). There are several reasons that may explain this situation. Approximately 85 out of the 195 member governments are developing economies and still use the Revised 1996 IPCC guidelines for reporting their emissions [17], which is in accordance with UNFCCC reporting requirements for developing countries. The revised 1996 IPCC guidelines pre-date the development of the managed land concept as a proxy to anthropogenic emissions and removals that was introduced in the 2003 IPCC Good Practice Guidance for Land Use, Land-Use Change and Forestry (LULUCF) [18], and incorporated into the 2006 IPCC Guidelines [9]. The latter documents require full representation of land use in the country, with all land being subdivided into one of the following land-use categories: forest land, cropland, grassland, wetlands, settlements and other land. In addition, forest land, wetlands and grassland must be divided into managed and unmanaged sub-categories, and although there are no GHG reporting requirements for unmanaged lands, countries are encouraged to report their areas. As more developing countries adopt the 2006 IPCC guidelines or even start using the 2003 LULUCF guidance, a complete representation of land use will need to be developed, and countries will have to determine which portions of their territory meet their definition of managed land.

Another 97 countries have adopted the later guidance from the IPCC [9, 18] that include a complete representation of land use, but have not provided information about their managed land base. It is not possible to determine the specific reasons for these decisions. Governments are not required to mention this concept in their national communication to the UNFCCC unless they have delineated a portion of the country as unmanaged, although application of the managed land proxy should be discussed based on IPCC Guidance [9, 18]. Regardless, all governments using the later guidance from the IPCC are implicitly using the managed land proxy, and many of these governments may consider their entire territory as managed land.

### Defining managed land

Among the governments providing information about application of the managed land proxy to subdivide forest land, grassland and wetlands into managed and unmanaged land, several have classified managed land simply by considering some land uses as managed and others as unmanaged. For example, Australia and Belarus consider all forest land, grasslands and wetlands as managed, while land in the ‘other land’ category (e.g., rock outcrops, glaciers, barren areas) is considered unmanaged. New Zealand considers all grassland and forest land as managed, while wetlands are considered unmanaged. The Russian Federation, Ukraine and Peru use the same criteria as New Zealand, except that they have designated some forest land in reserves or natural forests as unmanaged.

Some governments apply the managed land proxy with additional criteria, such as Canada, Brazil and the United States. Canada has subdivided grassland, forest land and wetlands into managed and unmanaged areas. Grassland is considered managed if used for grazing domestic livestock, designated as a national park or is used for an expressed purpose, such as a military base [19]. Canada considers wetlands in which human intervention has directly altered the water table level as managed, and there are two types: peatlands drained for peat extraction and flooded land associated with the creation of reservoirs. For reporting GHG emissions and removals, managed forests include all forest land that is subject to management for timber and non-timber resources as well as other ecological services. Therefore, managed land includes the timber harvest land base and other areas, including parks and reserved areas. Moreover, because fire suppression efforts in Canada also affect GHG emissions and removals, areas that are under intensive protection against natural disturbances, mostly fire, are considered managed forests even if there is no timber extraction or other non-timber forest use (Fig. 3).

Brazil defines all secondary forests and grasslands, planted forests, and forests subject to selective logging as managed land. Natural grasslands and natural forest land are considered unmanaged except in the following cases: [1] legally created Conservation Unit areas in Federal and State Conservation Units (Except the Private Reserves of National Preservation, due to the lack of consistent information regarding management interventions), in accordance with the Ministry of the Environment and the National System of Nature Conservation Units; and [2] legally demarcated Indigenous Lands, as per information from the National Indian Foundation (FUNAI). Managed wetlands include all reservoirs, which are created through human activity, while rivers and lakes are considered unmanaged. Other land is classified as unmanaged.

The United States has taken yet another approach by focusing on direct intervention [20]. The definition states “managed land occurs mostly in areas accessible to human activity and includes altering or maintaining the condition of the land to produce commercial or non-commercial products or services; to serve as transportation corridors or locations for buildings, landfills, or other developed areas for commercial or non-commercial purposes; to extract resources or facilitate acquisition of resources; or to provide social functions for personal, community or societal objectives where these areas are readily accessible to society” [20]. The United States government has incorporated accessibility of the land as a criterion, and included specific functions in the definition.

### Implementation criteria and application to delineate managed land

Implementation criteria are specific rules that are used to delineate managed land. For most of the countries, the implementation criteria are based solely on the land use maps developed to support the national GHG inventory. Governments are typically considering a land-use category as completely managed or not managed based on these criteria. Brazil, Canada and the United States provide examples for delineating managed land that includes additional criteria, subdividing individual land uses into both managed and unmanaged areas. The sections below provide further detail on the criteria and implementation methods for each country.

#### Canada

The proportion of the area of managed forests varies among the ten provinces and three territories in Canada. Some provinces, including British Columbia, Alberta, New Brunswick, Nova Scotia, and Prince Edward Island consider all forest land as managed due to ongoing timber harvest, fire protection and conservation activities. In contrast, Saskatchewan, Manitoba, Ontario and Quebec delineate a northern border of managed forests based on the northern boundary of fire suppression. In these provinces, areas to the north of this boundary are considered unmanaged. The Province of Newfoundland and Labrador includes all forests on the island of Newfoundland as managed but limits managed forests in Labrador to those that are subject to ongoing or planned future timber harvest. Yukon and the Northwest Territories delineate managed forest as areas designated for timber harvesting and areas under fire protection surrounding communities. Nunavut does not contain any managed forest. The initial delineation of managed forests was based on GIS boundaries of forest management units used for timber supply planning, parks and fire protection zones.

Managed forest areas can occasionally change over time if management activities expand into areas currently considered unmanaged forests. Managed forest lands can only leave this category through land-use conversion. Once forests are considered managed, they remain in that reporting category. Unmanaged forests can become managed forests if forest road construction and timber extraction commence, or if they meet other managed forest criteria. Unmanaged forests are monitored through Canada’s comprehensive deforestation monitoring program to ensure that resource extraction (mining), road construction and reservoir flooding events, which cause conversion of forest to non-forest land uses, are identified and the resulting emissions reported in the National GHG Inventory [12, 21].

The location and extent of cropland and grassland within a reporting unit is derived from remote sensing data [22]. Grassland is classified as managed within reporting units that have soil and climate conditions suitable for natural grassland and in which the Census of Agriculture identifies that agricultural activity is present [23]. All other mapped grassland is considered unmanaged.

The Atlas of Canada is used to determine the spatial extent of wetlands based on an inventory of wetlands, including those covered by forest at a national scale [24]. The Canadian Sphagnum Peat Moss Association has provided a map showing the principal peat harvesting areas [25] and an estimate of the wetland area managed for peat extraction in 2004 [19]. Estimates of the area under peat extraction for previous and subsequent years are made by adjusting the 2004 value on the basis of peat production. Flooded lands are delineated based on (1) forest conversion mapping [21], (2) the Canadian Reservoir Database [26], and (3) hydroelectric industry consultations [19]. Emissions from reservoirs are reported only for the 10 years following impoundment, and all flooding events that have occurred since 1980 are considered managed wetlands.

Settlements are identified through remote sensing data and population density mapping using Census data. A combination of the two approaches is applied because remote sensing data may lead to misclassification of large areas of trees or grass within an urban boundary, while population density mapping may miss isolated areas of manufacturing, transportation or resource extraction. Delineation of the settlement area is accomplished using the boundaries of Statistics Canada’s ‘Populated Centers’ [27] with populations over 30,000, which captures all major Canadian cities and represents 76% of Canada’s population. The total urban area for years prior to digital boundary availability was estimated using boundaries adjusted by manual editing of appropriate remote sensing data.

#### Brazil

Brazil applies IPCC approach 3 methods for land representation, meaning that classification is spatially explicit (i.e., all land area is explicitly classified). The Brazilian government utilizes several criteria (i.e., information plans/layers) and datasets to determine the managed land base, including biome maps, municipal boundaries, native vegetation maps (phytophysiognomy), soil types and land use/cover maps from visual interpretation of Landsat-type satellite imagery. The integration of all these georeferenced layers generates uniquely defined polygons in a Geographic Information System (GIS) that cover the entire national territory. Specifically, each polygon belongs to a single biome, municipality, vegetation type, soil type, and land use/cover. The native forest land and native grassland that fall over legally created Conservation Units and/or demarcated Indigenous Land are included as part of the managed land database. From one inventory year to another, changes in land use/cover are identified, and a polygon in one inventory year may be split into several sub-polygons in the next inventory year, if the entire polygon does not remain in the same land use/cover category. All the native forest land and grassland that is part of the managed land base and is subject to land clearing during the period of inventory is re-classified as another land use category in the same period, even in the case that the land could be temporarily unstocked with trees in the case of forest land.

#### United States

The United States has incorporated spatial data on specific anthropogenic functions to delineate the managed land base for the national GHG inventory [20]. These functions are based on the following criteria. All forest lands with active fire protection and timber harvesting are considered managed. Other anthropogenic activities occur on forest lands, but are within areas already managed for timber harvest or fire protection, or are converted to another land use based on the activity or change in land cover. All grasslands are considered managed at a county scale if there are livestock in the county. Other grassland and forest land areas are considered managed if accessible based on the proximity to roads and other transportation corridors, and/or infrastructure. Wetlands are considered managed if the water level is artificially changed, or the wetlands are created by human activity. Protected lands maintained for recreational and conservation purposes are considered managed (managed by public and private organizations). Lands with active and/or past resource extraction are considered managed.

Overall, the implementation criteria provide a link between the definition and the specific data that are needed to delineate the managed land base. Criteria are implemented using remote sensing imagery that has been classified into land uses. Lands that are used for crop production or settlements are all assigned as managed using the National Land Cover Dataset (NLCD) for the United States [28–30]. Active fire management is determined from maps of federal and state management plans from the National Atlas [31] and Alaska Interagency Fire Management Council [32]. Forest lands managed for timber harvests are informed by county level estimates of timber products reported in the U.S. Forest Service Timber Products Output Reports database [33]. Lands maintained for recreational purposes are determined from analysis of the United States Protected Areas Database [34] in which lands are classified as managed if there is suppression of natural disturbances or that are used for extractive or recreational purposes. Multiple data sources are used to delineate areas with active resource extraction, including the Alaska Oil and Gas Information System [35], Alaska Resource Data File [36], Active Mines and Mineral Processing Plants [37], and Coal Production and Preparation Report [38]. A buffer of 3300 and 4000 m is applied around petroleum extraction and mine locations, respectively, to account for the footprint of operations and impacts of activities on the surrounding landscape. Accessibility is a criterion unique to the United States among these three countries, and is a key determinant of managed area in remote areas of Alaska. Accessibility is determined using a 10 km buffer surrounding road and train transportation networks with the ESRI Data and Maps product [39], and a 10 km buffer surrounding settlements using the NLCD.

### Managed land bases in Canada, Brazil and United States

**Fig. 2:**
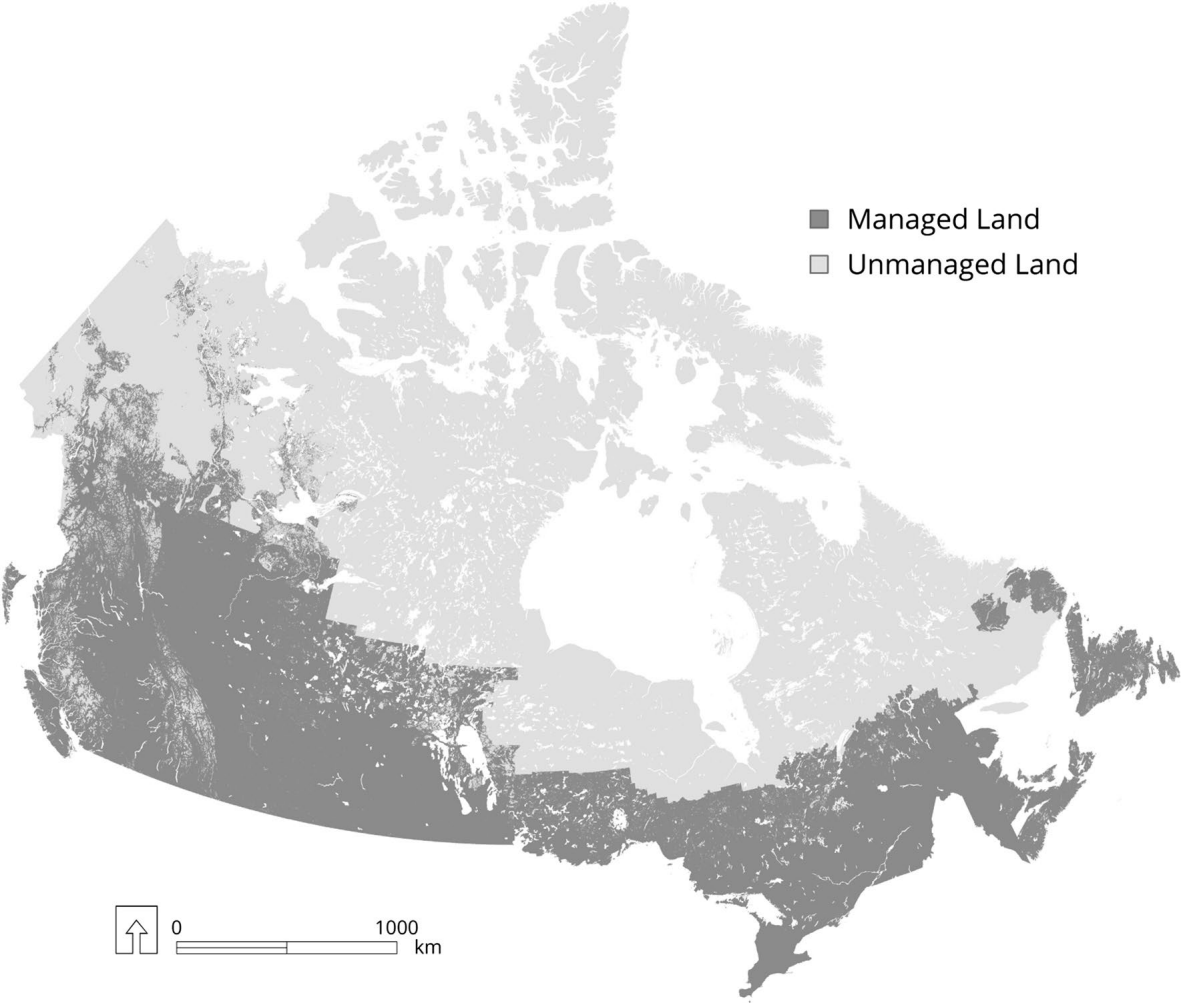
Distribution of managed and unmanaged land in Canada. The light gray areas are unmanaged and the darker gray areas are managed

The managed forest land in Canada in 2015 includes 225.9 Mha (Table 1) and represents 81% of the total managed land base of the country [40]. The total area of unmanaged forest in Canada is 118 Mha and all of this area is in northern regions of the country with limited or no access (Fig. 2). Canada also reported 45.1 Mha of cropland (16.1% of total managed land area) across 10 provinces and 6.6 Mha of managed grassland (2.4%) in Saskatchewan, Alberta and British Columbia. Settlements accounted for 0.9 Mha (0.3%) of the managed land base, and managed wetlands accounted for 0.5 Mha (0.02%). There are 512.4 Mha of unmanaged land in grasslands, wetlands and other lands, predominantly in northern Canada [41].

**Table 1:** Managed and unmanaged land in Brazil, Canada and the United States (millions of hectares)

Country	Managed land area	Unmanaged land area	Total land area
Canada	279.0	630.4	909.4
Cropland	45.1	0.0	45.1
Grassland	6.6	Unknown^a^	Unknown^a^
Forest land	225.9	118.0	343.9
Settlements	0.9	0.0	0.9
Wetlands	0.5	Unknown^a^	Unknown^a^
Other lands	0.0	Unknown^a^	Unknown^a^
Brazil (observed)	499.4	316.1	815.5
Cropland	68.5	0	68.5
Grassland	188.3	41.1	229.4
Forest land	235.3	258.3	493.6
Settlements	3.9	0	3.9
Wetlands	3.4	16.1	19.5
Other lands	0	0.6	0.6
Cloud-covered areas			36.7
United States	890	46	936
Cropland	159.2	0	159.2
Grassland	320.6	25.8	346.4
Forest land	292.7	9.6	302.4
Settlements	50.6	0	50.6
Wetlands	43.0	0	43.0
Other lands	24.7	10.8	34.5

^a^The total area of unmanaged wetland, grassland and other land is 512.4 Mha, but these areas are not disaggregated into the individual categories

Brazil has approximately 500 million hectares of managed land and 316 million hectares of unmanaged land based on an analysis for 2010 (Table 1) [42]. Brazil reports the net emissions for each one of its six biomes (Amazonia—AM; Cerrado—CE; Caatinga—CA; Mata Atlantica—MA; Pampa—PA; Pantanal—PT), with percent coverage of approximately 49, 24, 10, 13, 2 and 2% of the national territory (852, 187, 545 ha) respectively. In 2010, unmanaged native forest land corresponded to approximately 31% in AM; 30% in CE; 48% in CA; 16% in MA, 12% in PA and 54% in PT. Unmanaged native grassland corresponds to approximately 1, 13, 1, 2, 16 and 21% of the total territorial area of AM, CE, CA, MA, PA and PT, respectively. The percent coverage of other land (classified as unmanaged) in the total biome territorial area is near zero in all biomes except for PA, where it is approximately 1%.

**Fig. 3:**
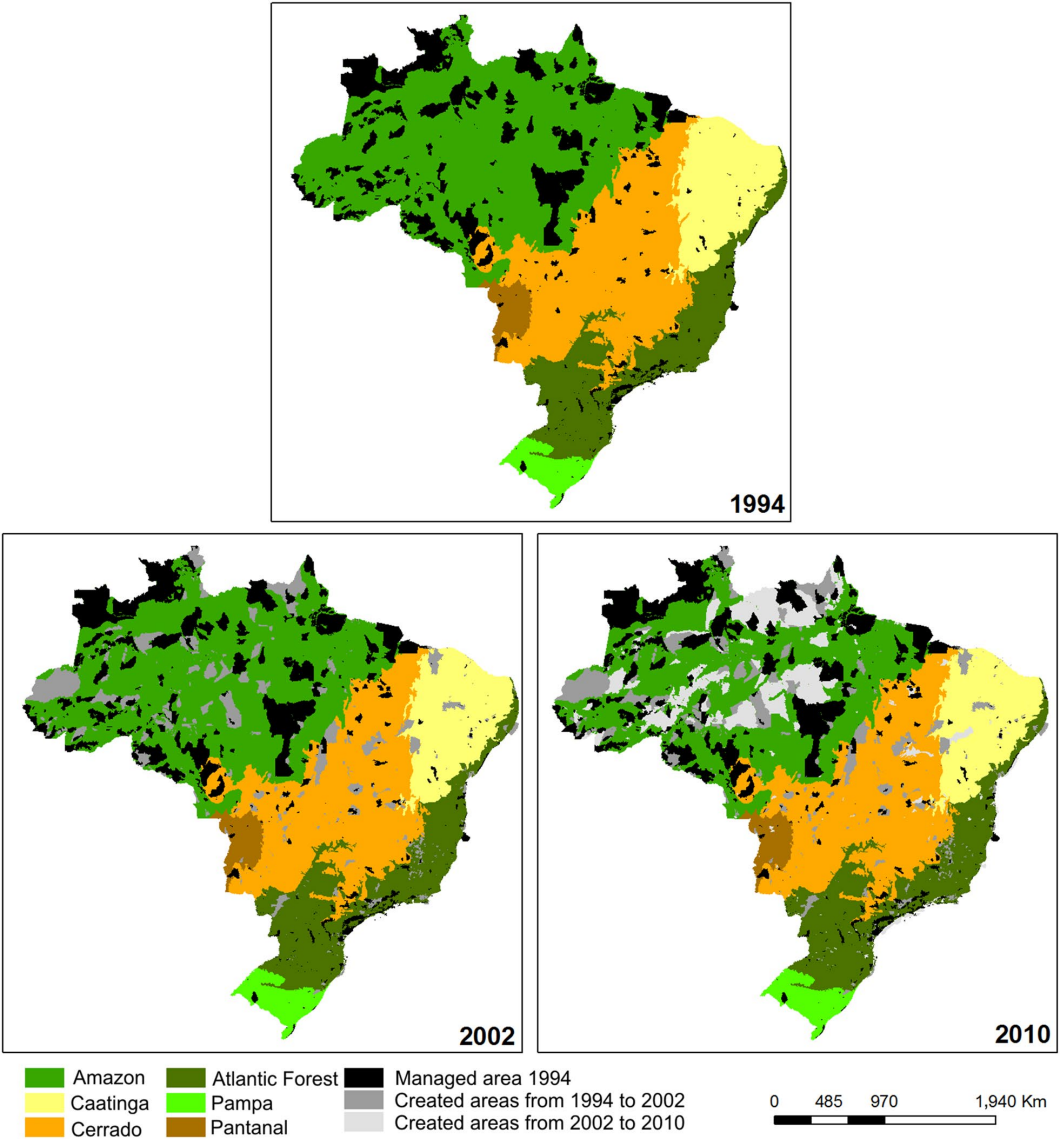
Distribution of the Conservation Units and Indigenous Lands in the Brazilian territory, by biome, as represented in the Third National GHG Inventory for 2002 and 2010. Biomes include Amazonia (intermediate green), Caatinga (yellow), Cerrado (orange), Pantanal (brown), Mata Atlantica (darker green), and Pampa (light green). In black, managed areas in 1994; in medium grey (areas created between 1994 and 2002); and light grey (areas created between 2002 and 2010)

Figure 3 shows the distribution of the Conservation Units and Indigenous lands (considered as part of the managed forest land) in the Brazilian territory, where their expansion from the periods 1994 to 2002 and 2002 to 2010 have been observed. The largest expansion occurs in the Amazonia biome, where most of the unmanaged forest land, grassland and wetlands also occurs (46.7%), followed by the Cerrado biome (27.9%). Approximately 36 million ha of land was cloud covered, corresponding to approximately 4% of the national territory [42].

**Fig. 4:**
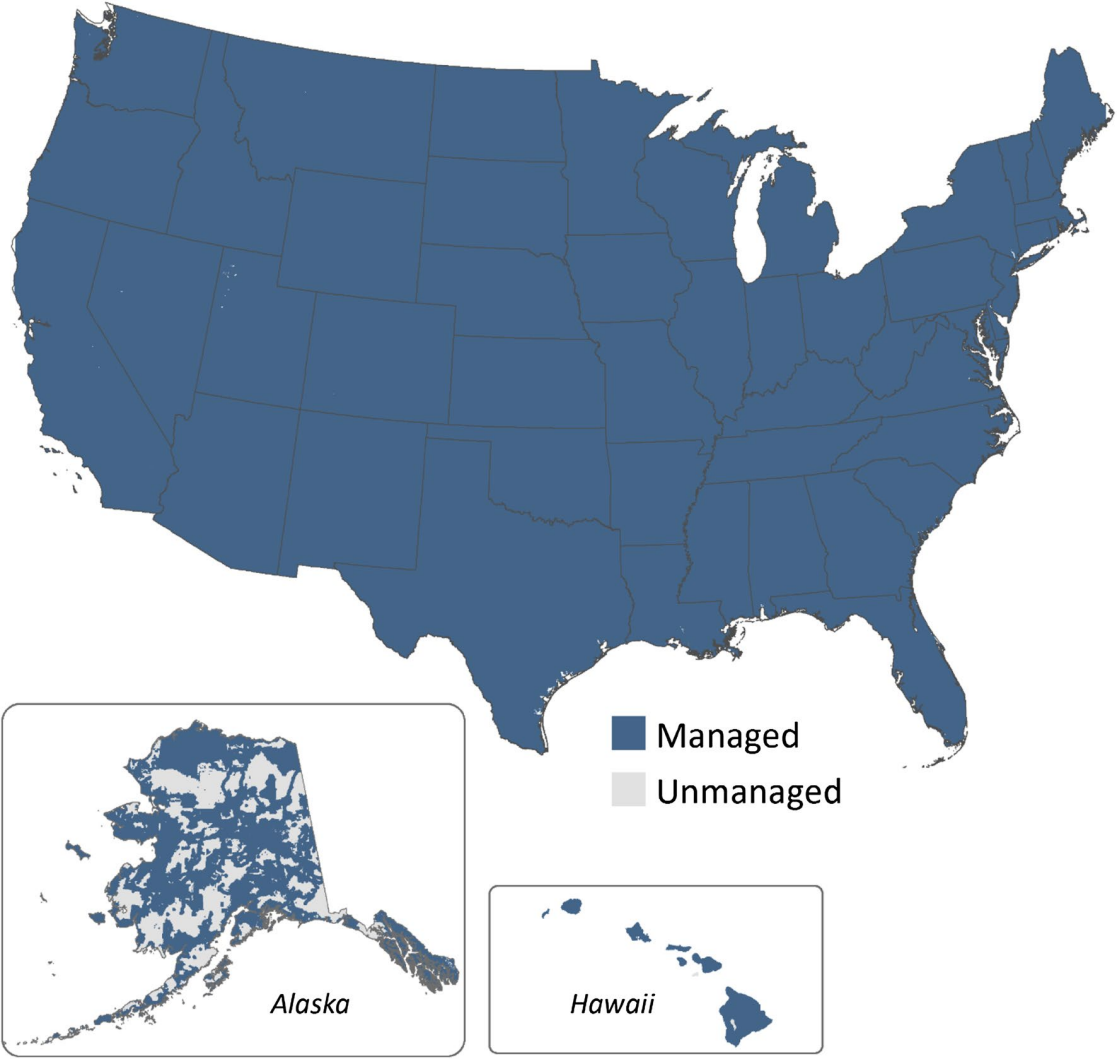
Distribution of managed and unmanaged land in the United States. The gray areas are unmanaged and the blue areas are managed

The total managed land base in the United States is 890 million hectares, and about 46 million hectares are unmanaged (Table 1, Fig. 4) [20]. Managed land includes the majority of the territory in the conterminous 48 states and Hawaii, where much of the land is actively managed for crop production, grazing livestock, timber production, or occurs within settlement areas. Moreover, the majority of the land in these regions is within a 10 km buffer of the road network, railroad corridors or settlements. There are large blocks of land in Alaska that are classified as unmanaged, which are grassland, forest land or wetland. These areas are largely inaccessible, which means that they are outside the 10 km buffer of road networks or other transportation corridors, and have no direct human management or function in which anthropogenic activities are influencing GHG emissions.

## Discussion

Two general approaches have been used to classify land as managed by countries reporting GHG emissions and C stock changes to the UNFCCC. The first approach is based on subdividing managed and unmanaged land according to the land-use classification with specific land uses designated as unmanaged. The second approach requires data on accessibility, vegetation structure and/or functional activities, such as grazing, mining, timber harvest, fire protection, crop production, conservation and social functions. Both approaches are in compliance with the reporting requirements established in the IPCC guidance [9–11], but arguably the second approach may allow national compilers to better discriminate between anthropogenic and non-anthropogenic emissions and removals.

Examples of the first approach include New Zealand that considers all wetlands as unmanaged, in addition to Australia and Belarus that consider land classified as “other lands” as unmanaged. Identifying some land uses as managed and others as unmanaged is a relatively simple and transparent approach. Lands are classified into land uses based on land cover maps derived from remote sensing imagery and other information. The land uses are expected to reflect specific functional activities, and therefore align reasonably well with areas that are managed and unmanaged in some countries.

The second approach incorporates additional data on specific functional activities, such as the approaches applied in Brazil, Canada and the United States. This approach is likely to be advantageous if land cover data do not align with functional activity. For example, forest land may not be managed in some parts of a country due to remoteness, lack of access, low human population density, or limited development in the region. Including these areas in the inventory would lead to unnecessary use of resources to compile information that is needed to estimate C stocks and associated changes, rather than focusing the time and effort in areas that are directly influenced by human activity. Furthermore, governments could over- or under-estimate anthropogenic emissions by conducting inventories for areas that are not managed.

Brazil, Canada, and United States have used the second approach to delineate their managed land areas, but even with these applications, each country is using elements of the first approach. Specifically each country classifies all cropland and settlements as managed, which is intuitive given that these areas would not even exist without direct human intervention. Forest lands, grasslands and wetlands incorporate functional or locational criteria to varying degrees to determine the managed and unmanaged land areas. For example, Canada and the United States determine the area of managed forest land based on timber harvest and extraction of other resources, active fire suppression and areas used for specific conservation and ecological functions. The government of Brazil uses similar criteria and includes in the managed land base, natural forest land and natural grassland in Conservation Units and/or Indigenous Lands, secondary vegetation (both in forest land and grassland), planted forests, and forests subject to selective logging. The United States also introduces a criterion of accessibility that leads to some remote areas, particularly in Alaska, being classified as unmanaged.

Grasslands are one of the more difficult areas to classify as managed or unmanaged because human intervention, particularly livestock grazing, can be difficult to ascertain from satellite remote sensing data. For example, even with clear implementation criteria, the identification of managed grassland in Canada is problematic with relatively low classification accuracy of 55, 58 and 69%, while in contrast, Canadian cropland has been mapped at 71, 82 and 93% accuracy for 1990, 2000 and 2010, respectively [22]. The relatively low accuracy of imagery classification is due to the similarity in reflectance between ‘native’ grassland that is used for livestock grazing and permanent pasture. Permanent pasture is land that has been improved by land clearing, stone removal, fertilization, breaking and seeding or fencing, but the level of improvement often declines over time to the point where the vegetation is similar to native grassland. With similar grazing practices, the distinction between the two is difficult, even with on-site visits.

The United States has attempted to circumvent some of these problems by classifying all grassland as managed in counties (smallest political unit in which Census data are available) that are known to have active livestock grazing based on Census data. However, this approach may lead to an over-estimation of managed grasslands, particularly in the western United States where there are large areas of native grasslands that may not be actively managed. Furthermore, even if livestock are active on these native grasslands, the impact on the structure of the vegetation may be limited compared to unmanaged grasslands, and so there may be little or no anthropogenic impact on C stocks and GHG emissions. Brazil uses a similar criterion to classify Protected Areas (or Conservation Units) and/or Indigenous Lands as managed, with the rationale that creating specific legislation is leading to a direct anthropogenic action. Similar to grasslands in the western United States, this policy may not have much impact on areas designated as Protected Areas and Indigenous Lands of Brazil. Implementing an additional criterion related to impacts on vegetation structure may save resources and time in conducting GHG inventories, by further focusing the estimation on those areas subject to anthropogenic emissions and removals.

Non-anthropogenic emissions can occur on managed land, and can be problematic for reporting anthropogenic emissions to the UNFCCC. For example, Brazil reports the area and associated CO_2_ emissions from natural forest land previously classified as unmanaged when it becomes part of the managed land base. From 2002 to 2010, there were 59 million ha of new managed land, mostly in the Amazonia region, and these areas sequestered 190 Tg CO_2_. Indirect human-induced effects, such as CO_2_ fertilization and/or N deposition, are most likely driving the net uptake of CO_2_ in these areas, and consequently the sinks are not under the direct control of anthropogenic management activity. It may be possible to determine the non-anthropogenic emissions from the managed land base although this is not trivial as noted previously [15, 43]. These methods could help national inventory compilers to disaggregate the reporting into those emissions and removals that are clearly the result of human activities, and those that are the results of indirect anthropogenic activity or other emissions drivers, such as natural disturbances resulting from insect outbreaks and wildfires. For example, in their 2017 National GHG Inventory, Canada reported separately the emissions and removals from forest lands that have been subjected to natural disturbance and all emissions and removals on managed forest lands [40]. Indirect effects of anthropogenic activity, such as increased wildfires or pest and disease outbreaks that lead to increased flux of CO_2_ to the atmosphere, or CO_2_ fertilization that increases net primary production and removals, in principle should not be included in the reported estimates because they are not directly under the control of land managers. However, aside from the attempts to disaggregate emissions and removals from natural disturbances by Canada and Australia, factoring-out has not been attempted in any other National GHG Inventory, and more evaluation is needed to determine the effectiveness of these approaches.

The exclusion of unmanaged lands may lead to scientifically incomplete understanding of the greenhouse gas fluxes between the land surface and atmosphere. For example, much of the unmanaged land areas in Canada and Alaska contain deep organic layers and permafrost that are susceptible to a range of climate change impacts from thawing, water table changes if ice melt allows water to drain, and wildfires [12, 44]. The response of these lands to climate change and the associated emissions could have significant impacts on the global C cycle (both CO_2_ and CH_4_). While it would not be appropriate to report these as anthropogenic emissions, the fluxes can have important implications for global policies aimed at achieving GHG reduction targets or atmospheric CO_2_ concentration targets. If emissions from unmanaged forests, peatland or permafrost C (positive feedback) use up some of the remaining “allowance” for C emissions to the atmosphere [45], then mitigation efforts in all other sectors have to increase to meet the global CO_2_ reduction targets. While currently there are no UNFCCC requirements for monitoring and reporting of GHG emissions from these areas by governments because they are designated as unmanaged, including these areas could contribute more certainty to the outcomes of policy programs intended to limit the impact of anthropogenic GHG emissions on the climate system.

According to the IPCC, it is good practice to be transparent about the methods that are used for estimating and reporting GHG emissions [9]. Based on our review, governments could be more transparent about their application of the managed land proxy in national communications. Many communications do not explicitly mention managed land even though the concept is implicitly applied by the 97 government that are using the later guidance from the IPCC [9, 18] based on our review. Specifically, governments could provide a definition for managed land and implementation criteria. If some of the land is not managed, then it would also be useful to provide a map with the spatial distribution of managed and unmanaged land. In addition, changes in the managed land base over time should be reported, and the effect of those changes on emission and removals.

## Conclusion

Delineating a managed land base is the only universally accepted approach for estimating anthropogenic emissions and removals associated with land use [14]. The managed land proxy has weaknesses because it is not feasible to fully discriminate between anthropogenic and non-anthropogenic emissions and removals. Non-anthropogenic emissions and removals from processes such as natural disturbances, nitrogen deposition, and CO_2_ fertilization do occur on managed land, and are difficult, if not impossible, to separate from anthropogenic sources. Regardless, there is flexibility in the application of the proxy to meet national circumstances as illustrated in the examples from Brazil, Canada and the United States, by incorporating additional criteria to better delineate the managed land base, such as functional criteria, vegetation structure, and accessibility. In turn, this will focus attention on the regions that are influenced directly by management activity with the goal of developing effective GHG mitigation policies enhancing land-based sinks and/or reducing sources of emissions by improving land management.

## Abbreviations

C: carbon
IPCC: Intergovernmental Panel on Climate Change
UNFCCC: United Nations Framework Convention on Climate Change
